# Oxidative stress alters global histone modification and DNA methylation

**DOI:** 10.1016/j.freeradbiomed.2015.01.028

**Published:** 2015-02-03

**Authors:** Yingmei Niu, Thomas L DesMarais, Zhaohui Tong, Yixin Yao, Max Costa

**Affiliations:** 1 Occupational Disease and Toxicology Department, Beijing Chao-Yang Hospital, Capital Medical University, Beijing, China 100020; 2 Department of Environmental Medicine New York University School of Medicine, Tuxedo, New York 10987, USA; 3 Department of Respiratory and Critical Care Medicine, Beijing Institute of Respiratory Medicine, Beijing Chao-Yang Hospital, Capital Medical University, Beijing, China 100020; 4 Department of Biochemistry and Molecular Pharmacology New York University School of Medicine, New York, New York 100016, USA; 5 Shanghai Jiao-Tong University School of Public Health, Shanghai, China 200025

**Keywords:** oxidative stress, histone demethylase, hydrogen peroxide

## Abstract

The JmjC-domain-containing histone demethylases (JHDMs) can remove histone lysine-methylation and thereby regulate gene expression. The JmjC-domain uses iron Fe (II) and α-ketoglutarate (αKG) as cofactors in an oxidative demethylation reaction via hydroxymethyl-lysine. We hypothesize that reactive oxygen species will oxidize Fe (II) to Fe (III), thereby attenuating the activity of JmjC-domain-containing histone demethylases. To minimize secondary responses from cells, extremely short periods of oxidative stress (3 hours) were used to investigate this question. Cells that were exposed to hydrogen peroxide (H_2_O_2_) for 3 hours, exhibited increases in several histone methylation marks including H3K4me3 and decreases of histone acetylation marks including H3K9ac and H4K8ac; pre-incubation with ascorbate attenuated these changes. The oxidative stress level was measured by generation of 2′, 7′-dichlorofluorescein (DCF), GSH/GSSG ratio and protein carbonyl content. A cell free system indicated H_2_O_2_ inhibited histone demethylase activity where increased Fe (II) rescued this inhibition. TET protein also showed a decreased activity under oxidative stress. Cells exposed to a low dose and long term (3 weeks) oxidative stress also showed increased global levels of H3K4me3 and H3K27me3. However, these global methylation changes did not persist after washout. The cells exposed to short term oxidative stress also appeared to have higher activity of class I/II histone deacetylase (HDAC) but not class III HDAC. In conclusion, we have found that oxidative stress transiently alters epigenetic program process through modulating the activity of enzymes responsible for demethylation and deacetylation of histones.

## Graphical Abstract

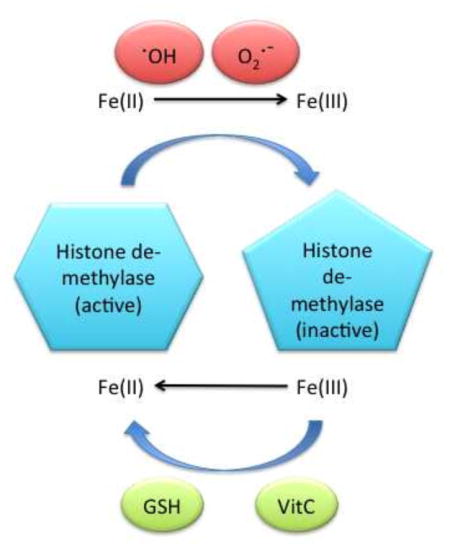

## Introduction

Reactive oxygen species (ROS) such as superoxide anion radical, hydrogen peroxide (H_2_O_2_) and hydroxyl radical are generated endogenously through mitochondria respiration as well as through exogenous oxidizing agents including ionizing radiation, heavy metals, and hypoxia. ROS affects a wide range of biological process through a variety of mechanisms. Different hypotheses have been proposed regarding the responses of the epigenetic machinery components such as histone modifying enzymes based on the availability of α-ketoglutarate (αKG) and ascorbate [1,2], but studies that directly investigate the effect of oxidative stress on such epigenetic modifying enzymes are rare in the literature.

Histone methylation is one of important epigenetic modification that can occur on several lysine or arginine residues, primarily on histone H3 and histone H4 [3]. Soon after it was first discovered, histone methylation was thought to be a stable modification, as no enzymes were known that could remove this mark [2]. Now, it is known that methylation of histone lysines can be removed by oxidative histone demethylase, including flavin adenine dinucleotide (FAD) dependent amine oxidase, and Fe (II) and αKG dependent dioxygenase [4, 5]. The JmjC-domain of Fe (II) and αKG dependent dioxygenase utilizes Fe (II) and αKG as co-factors in an oxidative demethylation reaction that produces hydroxymethyl-lysine [6]. *In vitro* studies have revealed that while some JmjC have quite particular selectivity for histone residues, such as KDM3A that is specific to mono- and di-methylation on histone H3 lysine 9 (H3K9); others have a broader range of targets [7].

Another pivotal epigenetic enzyme utilizing Fe (II) and αKG as co-factors is ten-eleven translocation (TET) family of hydroxylases. TET oxidizes 5-methylcytosine (5-mC) to 5-hydroxymethylcytosine (5-hmC), which has been recognized as “the sixth DNA base” [8]. 5-hmC can be further converted into 5-formylcytosine (5fC) and 5-carboxylcytosine (5caC) serving as intermediates in an active demethylation pathway that ultimately replaces 5mC with cytosine in non-dividing cells [9]. In addition DMT1 that replicates DNA methylation patterns during S phase does not recognize 5-hmC and this leads to passive demethylation of DNA. Currently, the generation of 5-hmC in mammalian cells is primarily attributed to enzymatic oxidation of 5mC by three isoforms of the TET protein (TET1-3) [8]. Since the oxidation of 5mC catalyzed by TET proteins requires several co-factors [Fe (II), αKG, and ascorbate] [8, 10], the catalytic activity of TET proteins should be affected by changes in the levels of these co-factors in mammalian cells.

ROS can be catalyzed by metals [11] and hypoxia [12] resulting in increases in histone methylation marks that were attributable to the inhibition of histone demethylase activity [13–15]. These global alterations include H3K4me2, H3K4me3, H3K79me3, H3K27me3 and H3K9me2 [13–15]. As an important ROS, H_2_O_2_ is produced in various physiological and pathological conditions. Normal human plasma typically contains 1–8 μM H_2_O_2_ [16,17]. Higher concentrations have been observed in activated macrophages, up to a local concentration as high as 1mM [18]. Elevations in H_2_O_2_ have been detected in numerous pathological conditions, including ischemia and reperfusion where an extracellular H_2_O_2_ concentration of 75–170 μM has been observed in the affected brain [19]. Respiratory lining cells in subjects with inflammatory lung disease typically experience 2–20μM H_2_O_2_ [20]. An important consideration is that the subcellular concentration of H_2_O_2_ can vary immensely and be much higher in an individual cell or subcellular compartment compared to a cell-wide average concentration [21].

In this study, we hypothesized that oxidative stress would cause a loss of reduced ascorbate, which was required to reduce Fe (III) back to Fe (II) and regenerate the active dioxygenase enzyme, after molecular oxygen is split during catalysis. The entire epigenetic program of the cell can be altered albeit temporarily, if these enzymes are inhibited. Three concentrations of H_2_O_2_ were chosen to mimic local high concentration within activated macrophage (250 μM, 3 hours), short-term ischemia and reperfusion (150 μM, 3 days) as well as long-term inflammation (25 μM, 3 weeks). Ascorbate was employed as a reactive oxidative species scavenger in the current study.

## Material and Methods

### Cell lines and antibodies

Immortalized human bronchial epithelial cells (BEAS-2B) were obtained from the American Type Culture Collection (Manassas, VA). Cells were cultured in DMEM supplemented with 10% fetal bovine serum (FBS) at 5% CO_2_. Transgenic V79 Chinese hamster G12 cells were grown in F-12 medium. Antibodies against total histone H3 and modified histones were purchased from Abcam (Cambridge, MA). Antibodies against 5-mC or 5-hmC were purchased from Zymo Research (Irvine, CA)

### Western blotting

After defined treatments, cells were extracted by colorless protein boiling buffer (62.5 mM Tris-HCl, pH 6.8, 4% w/v SDS, 10% glycerol, 50 mM DTT) immediately and boiled at 95°C for 5 minutes. Total protein was quantified with a DC protein assay kit (Bio-Rad). Proteins (20μg/lane) fractionated over 15% SDS denaturing gels (Bio-Rad) were transferred to PVDF membranes (Fisher) followed by immunoblotting. Proteins were transferred to PVDF membranes that were blocked with PBS +0.1% Tween 20 containing 5% nonfat dry milk. The membranes were then incubated overnight with primary antibodies using dilutions suggested by the manufacturers. The membranes were then incubated with secondary antibodies for 1 h at room temperature. After thorough washing with PBST buffer, signals on membranes were developed with an ECL system (Bio-Red). The intensity of the stained bands was then quantified by imageJ (Image Processing and Analysis in Java, NIH) and the relative quantifications were shown as numbers below the bands in the figures.

### ROS assays

Oxidative stress levels were measured by dichlorodihydrofluorescein (DCF) imaging, the ratio of reduced glutathione (GSH)/glutathione disulfide (GSSG) and protein carbonyl content assay. For DCF imaging, cells were incubated with 25μM 5-and-6-carboxy-2′,7′-dichlorodihydrofluorescein diacetate (carboxy-H2DCFDA) at 37°C for 30 minutes. Hoechst 33342 was added at a final concentration of 1.0 μM to the carboxy-H2DCFDA staining solution during the last 5 minutes of the incubation. The cells were then imaged by Leica TCS SP5 (Leica Microsystems, IL). For GSH/GSSG ratio, GSH/GSSG-Glo assay from promega (Madison, WI) was used according to manufacturer’s guide. Five thousand live cells were seeded in triplicates in white wall 96-well plates 8 hours before the treatment to allow the cells to attach, right after the treatment, cells were washed and lysed in the plate and GSH/GSSG ratio was measured by luminescence signal. Protein carbonyl content assay kit was purchased from Sigma Aldrich (St. Louis, MO), Carbonyl content was determined by the derivatization of protein carbonyl groups with 2,4-dinitrophenylhydrazine (DNPH) leading to the formation of stable dinitrophenyl (DNP) hydrazone adducts, which was then detected spectrophotometrically at 375 nm, proportional to the carbonyls present. The measurements were carried out in duplicates.

### H3K4 demethylation assay

Nuclear extracts were prepared using a CelLytic NuCLEAR extraction kit purchased from Sigma (St. Louis, MO). Freshly prepared nuclear extracts (50 μg) from BEAS-2B cells were incubated with 5 μg of histones purchased from Santa Cruz (Santa Cruz, CA) in histone demethylation buffer [50 mmol/L HEPES (pH 8.0), 2 μg/mL bovine serum albumin, 0.1 mmol/L DL-DTT, 100 μmol/L FeSO_4_, 2 mmol/L ascorbate, 1 mmol/L α-ketoglutarate, and protease inhibitors] in a final volume of 50 μL at 37°C. Following 1 hour incubation, the demethylation reaction was terminated by the addition of EDTA to a final concentration of 1 mmol/L(or 6 X protein loading buffer to stop reaction). The reaction mixture was analyzed by Western blotting using H3K4me3 antibody. The intensity of the stained bands was then quantified by imageJ and the relative quantifications were shown as numbers below the bands in the figures.

### Histone deacetylase (HDAC) activity assay

Class I/II HDAC activity was determined by Active Motif (Carlsbad, CA) colorimetric HDAC assay kit. nicotinamide was present in each reaction at 10μM to suppress potential Class III HDAC activity in nuclear extracts. Class III HDAC activity was determined by Promega (Madison, WI) SIRT-Glo assay system, trichostatin A (TSA) was present in each reaction at 1μM to suppress potential Class I/II HDAC activity. Linear range was first determined according to manufacturer’s guide, then 10ug of nuclear extract using CelLytic NuCLEAR extraction kit (Sigma) was used for Class I/II HDAC activity and 50ug was used for activity measurement.

### DNA ELISA assays

DNA methylation level was examined by 5-mC and 5-hmC DNA ELISA kit purchased from Zymo Research (Irvine, CA). TET protein activity was measured by epigenase 5mC-hydroxylase TET activity kit purchased from Epigentek (Farmingdale, NY). For DNA methylation level, the genomic DNA was first extracted utilizing QIAamp DNA kit purchased from Qiagen (Germantown, MD), 100ng DNA from each sample was then examined by 5-mC and 5-hmC DNA ELISA kit in triplicates according to manufacturer’s guide. For epigenase 5mC-hydroxylase TET activity, 10μg of nuclear extracts from each sample was examined in triplicates according to manufacturer’s guide.

### Methylation (5-hmC) dependant DNA immunoprecipitation

Genomic DNA was first extracted utilizing QIAamp DNA kit purchased from Qiagen (Germantown, MD), 500ng DNA from each sample was then diluted in DNA denaturing buffer (Zymo) fragmented into 200–400bp by sonication and denatured at 98°C for 5 minutes. In each immunoprecipitation (IP), 15μl protein A magnetic beads and 1.6 μg 5-hmC antibody was incubated immediately with the denatured DNA for 1 hour. After proper wash steps, the immunoprecipitated DNA was eluted into DNA elution buffer (Zymo) and subject to quantitative real-time PCR by ABI 7900 HT fast real-time PCR system (Life technology). Primers for gpt genes are as follows: B-fwd 5′-3′ GCACGTAAACTC, B-rev 5′-3′ TGGAAAGGCATTATTGCGCTAAGC; F-fwd 5′-3′ TTGCGATTCGTGAAATGTATCC, F-rev 5′-3′ AAACCGGCTGGTCGTCC; A-fwd 5′-3′ TGGGATATGGGCGTCGTATT, A-rev 5′-3′ AATCTTTCAACGCCTGGCA.

## Results

### Oxidative stress affects global histone modification levels

**Figure 1:**
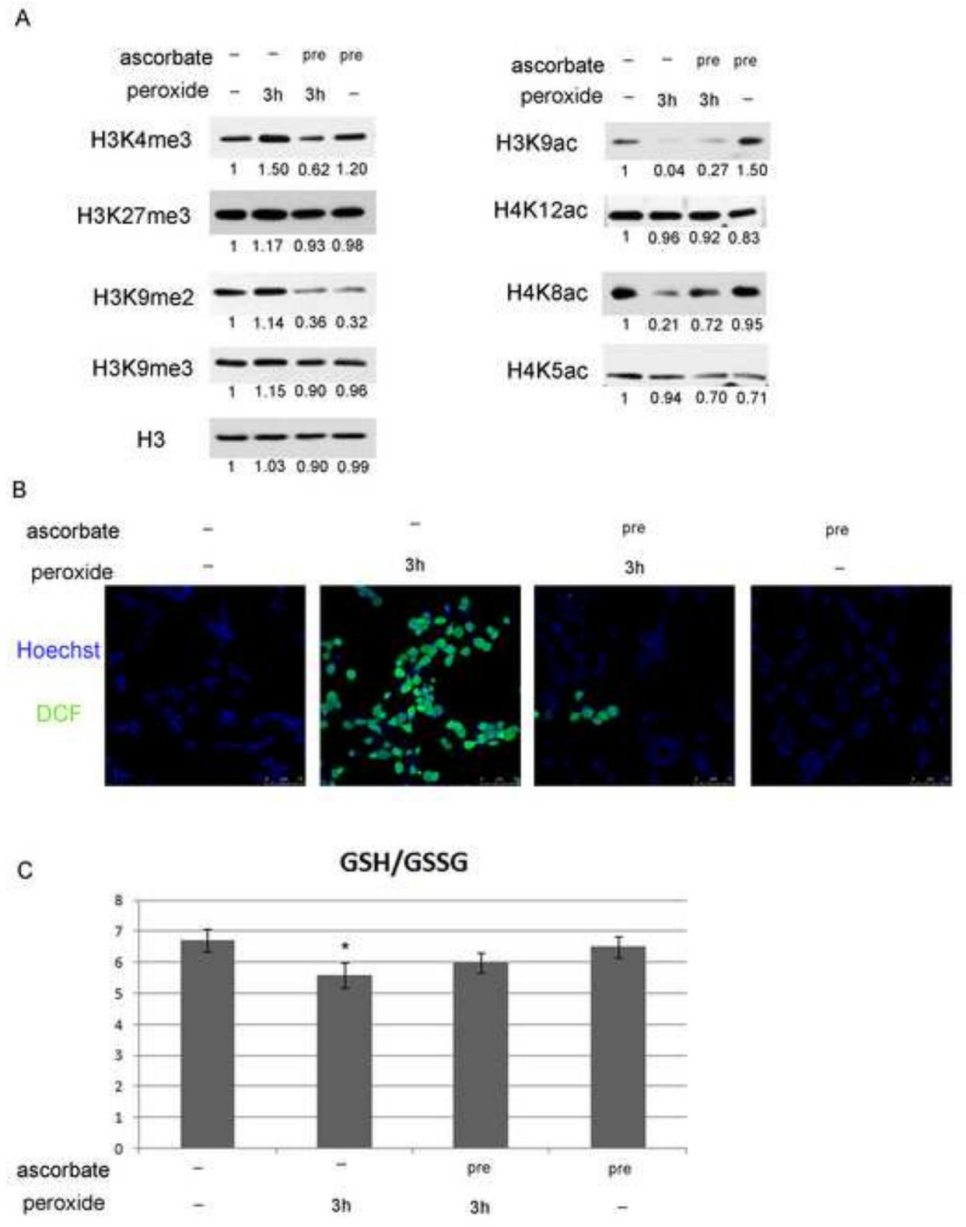
Global histone modification level in cells exposed to H_2_O_2_ for 3 hours with or without ascorbate pretreatment. (A)The ascorbate pretreatment was conducted at 250μM, 3 hours before sham or H_2_O_2_ was introduced to the BEAS-2B cell culture at 250μM for 3 hours. Histone H3 serves as a loading control. Band intensity relative to control band in each panel has been quantified and relative intensities are shown by the numbers below each panel. (B) Microscopy images of cells stained by carboxy-H2DCFDA. The cells were treated with 250 μM H_2_O_2_ for 3 hours, stained with carboxy-H2DCFDA (green) and Hoechst (blue); and observed using a confocal microscope. (C) GSH/GSSG ratio in cells exposed to H_2_O_2_ for 3 hours with or without ascorbate pretreatment.

To elucidate the potential effects of oxidative stress on histone demethylase, global histone methylation levels in BEAS-2B cells exposed to hydrogen peroxide (H_2_O_2_) with or without ascorbate pre-incubation (3 hours or 16 hours followed by thorough wash out right before the H_2_O_2_ exposure) were examined by western blot. The histone modification levels were evaluated in three independent experiments, a representative result with an estimation of band intensity by imageJ is shown in Figure 1. Global histone methylation of histone H3K4, K27 and K9 increased after cells exposed to H_2_O_2_, while a pre-incubation with ascorbate reduced this effect. (Figure 1A) The pretreatment of ascorbate alone followed by a sham exposure reduced global methylation of H3K9me2 (Figure 1A). In addition, global H3K9ac and H3K8ac acetylation levels were decreased by H_2_O_2_ (Figure 1B).

**Figure 2:**
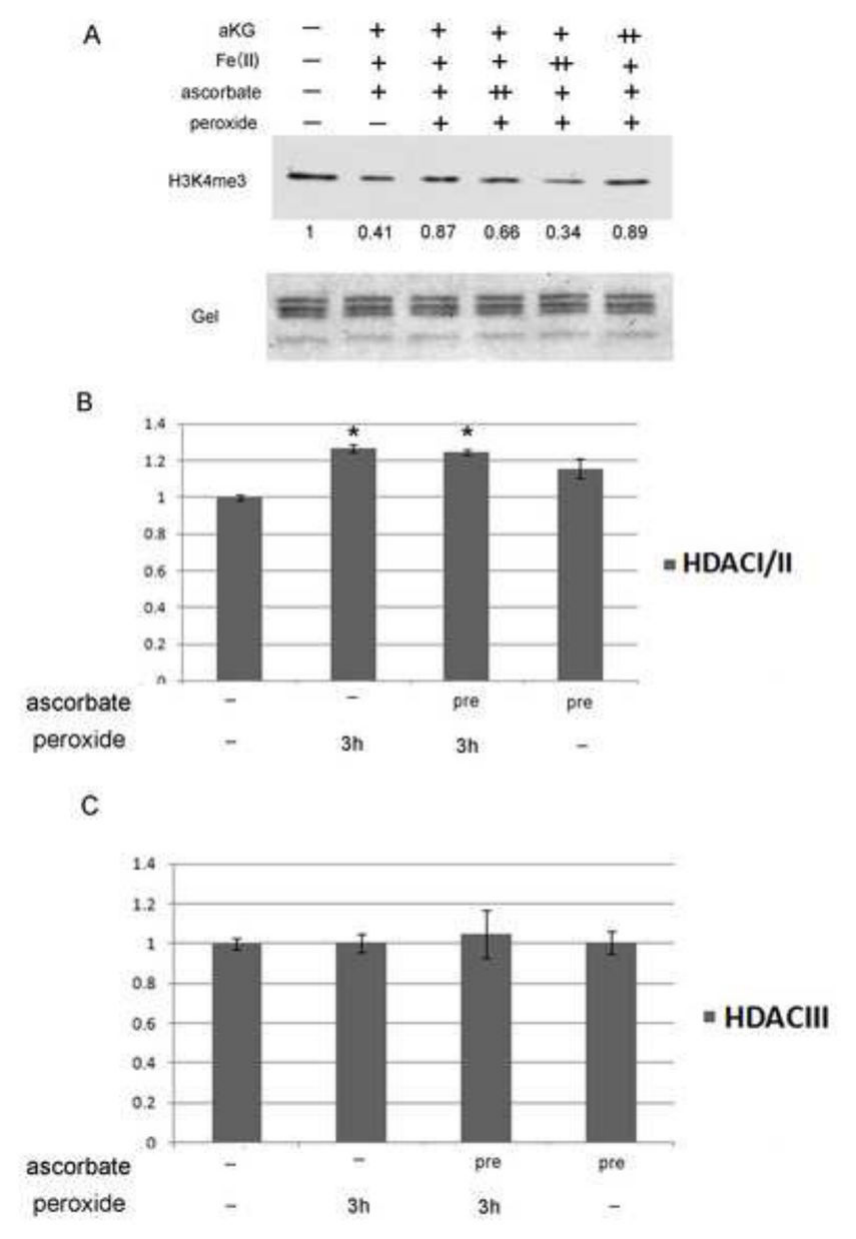
Oxidative stress modulates the activity of histone modifying enzymes. (A) H_2_O_2_ inhibited the activity of histone H3K4 demethylase *in vitro*. The histone H3K4 demethylation assay was performed as described in Materials and Methods. The reaction mixture was incubated at 37°C for 1 hour. The intensity of the bands was quantified, and relative values relative to control condition were shown. + denotes 250uM for H_2_O_2_, 100μM for Fe(II), 1mM for ascorbate and 2mM for αKG; ++ denotes 200μM for Fe(II), 2mM for ascorbate and 4mM for αKG. *p<0.05 The lower panel shows stained protein gel image as a loading control.(B),(C) HDAC activity in cells exposed to H_2_O_2_ for 3 hours with or without ascorbate pretreatment.

The oxidative stress level was measured by cell generation of dichlorodihydrofluorescein (DCF) fluorescence. As shown in Figure 2A, exposure to H_2_O_2_ induced copious amounts of intracellular hydrogen peroxide compared to controls. The pretreatment of ascorbate prevented the induction, albeit the intracellular hydrogen peroxide level was still higher than the control cells (Figure 2B). It is worth noting that the generation of DCF fluorescence relies on •OH production from H_2_O_2_ via Fenton reaction in the presence of Fe (II) [22], raising the concern that direct oxidation of Fe (II) to Fe (III) may reduce the precision of DCF fluorescence signal. Another classical method to study the consequences of oxidative stress is to measure the thiol-based redox couples such as reduced glutathione (GSH)/glutathione disulfide (GSSG) which is close linked to free radical status in cells [23]. To confirm the effect of oxidative stress, GSH/GSSG ratio was measured in cells immediately following the exposure. The H_2_O_2_ exposed cells showed a reduced ratio when compared to control cells, ascorbate pretreated cells and H_2_O_2_ exposed cells with ascorbate pretreatment, suggesting that H_2_O_2_ exposure introduced sufficient oxidative stress to oxidize GSH and ascorbate pretreatment attenuated this effect. The carbonylated protein content measurements also indicated the treatment induced sufficient oxidative stress to increase protein carbonyl content in the exposed cells compared to controls (Table 1).

### Oxidative stress modulates the activity of histone modifiers

To determine factors that compromised histone demethylase activity under oxidative stress (other than protein oxidation), a cell free system was employed. Since the global level of tri-methylation on H3K4 was increased to the greatest extent, this mark was used as a readout for the histone demethylase activity assay. H_2_O_2_ indeed inhibited histone demethylase activity and excessive amounts of Fe (II) and ascorbate rescued this inhibition while increased αKG did not, indicating oxidized iron was one of the factors that lead to lowered activity of histone demethylase. (Figure 2A)

Given that class I/II and III HDAC require different co-factors in their reaction, their activity was examined separately and an appropriate inhibitor was added to exclude cross-reaction. The class I/II HDAC showed an increased activity in the cells exposed to H_2_O_2_ with or without ascorbate pretreatment, while class III HDAC showed no significant change of its activity (Figure 2B–C).

### Global histone modification levels and their sustainability following long-term oxidative stress

**Figure 3:**
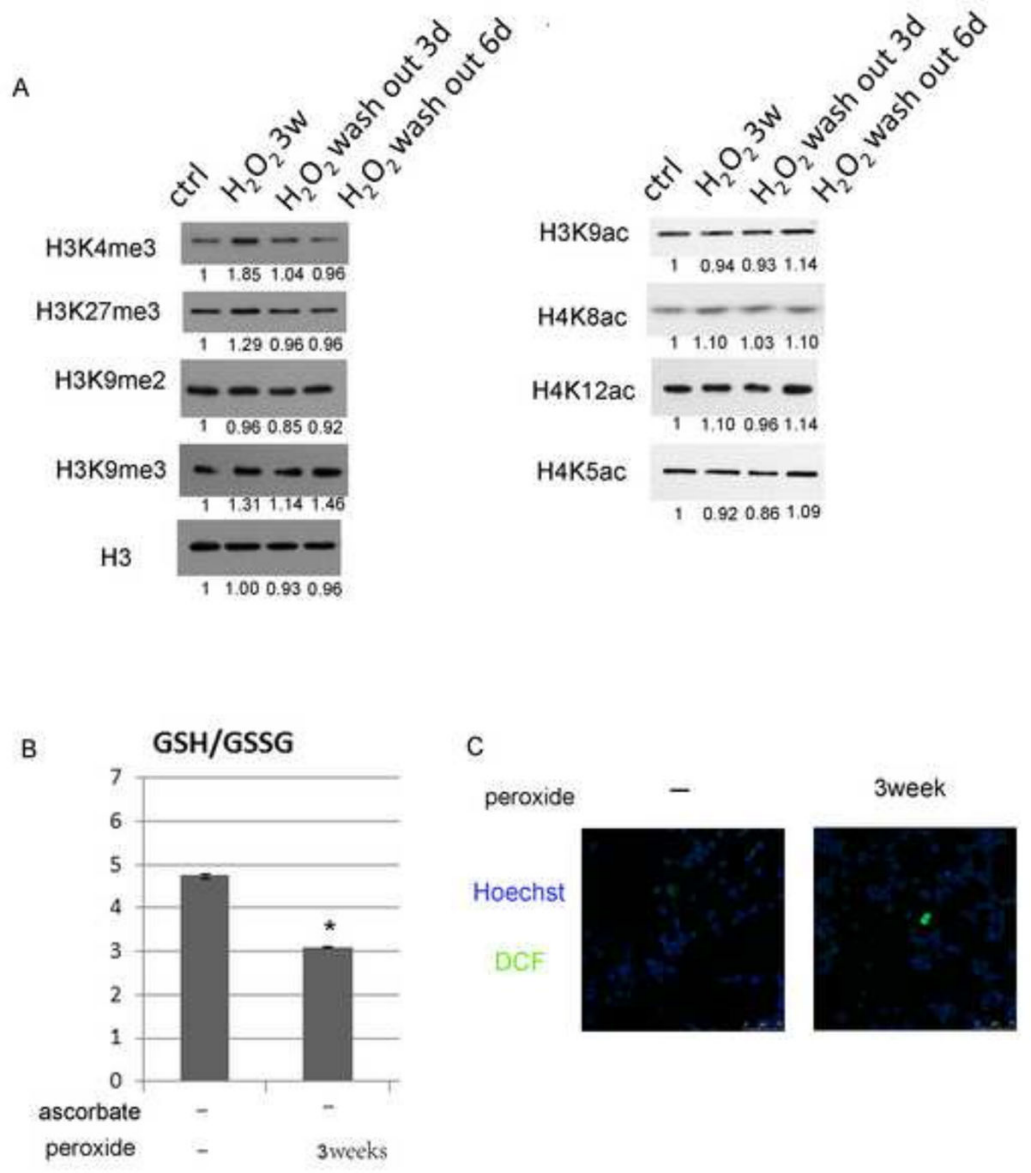
Global histone modification level in long term oxidative stress and sustainability. (A) Global histone modification level in cells exposed to H_2_O_2_ for 3 weeks. (B) GSH/GSSG ratio in cells exposed to H_2_O_2_ for 3 weeks, *p<0.05. (C) Microscopy images of cells stained by carboxy-H2DCFDA. The cells were treated with 25 μM H_2_O_2_ for 3 weeks, stained with carboxy-H2DCFDA (green) and Hoechst (blue); and observed using a confocal microscope.

**Figure 4:**
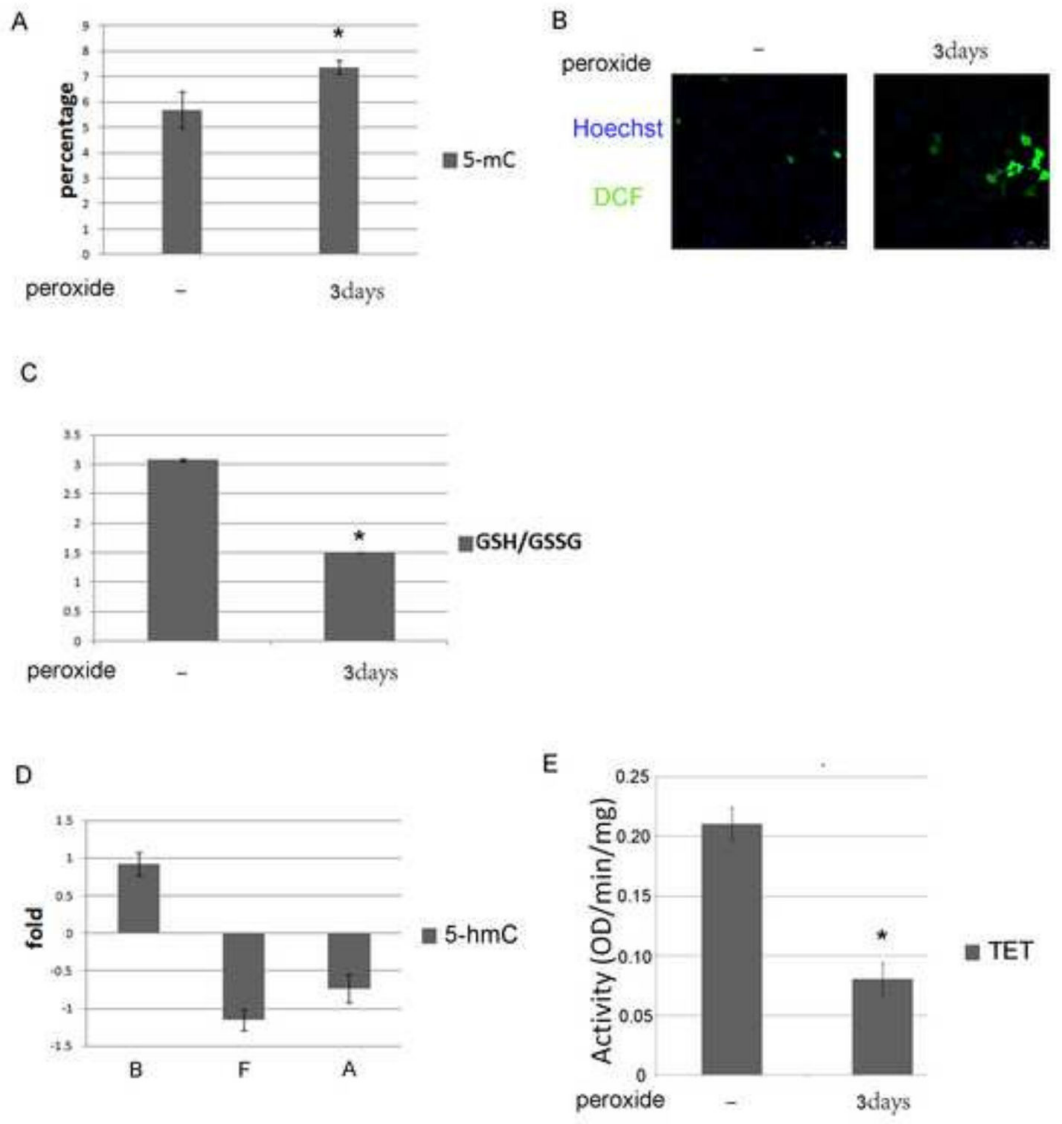
TET activity and DNA methylation under oxidative stress. (A)Global 5-mC level of BEAS-2B cells exposed to 150 μM H_2_O_2_ for 3 days. (B) Microscopy images of BEAS-2B cells stained by carboxy-H2DCFDA. The cells were treated with 150 μM H_2_O_2_ for 3 days, stained with carboxy-H2DCFDA (green) and Hoechst (blue). (C) GSH/GSSG ratio in BEAS-2B cells exposed to H_2_O_2_ for 3 days. (D). Fold enrichment of different areas of *gpt* gene after 5-hmC dependent DNA IP in G12 cells exposed to 150 μM H_2_O_2_ for 3 days when compared to control G12 cells. (E)TET activity in G12 cells. *p<0.05

To address the question of whether these phenomena observed during short term H_2_O_2_ exposure were also present following long term oxidative stress, cells were exposed to lower doses (25μM H_2_O_2_, which yielded a survival rate of more than 50% following 3 weeks of exposure). Whole cell lysate was collected after the treatment and also after a 3-day or 6-day wash out time interval. Global histone modification levels were assessed by western blot and the intensities of these western blots were quantified (Figure 3A and 3B). Although several histone methylation marks showed increases after long-term oxidative stress, the alteration did not persist after wash out (Figure 4A). The global histone acetylation level did not appear to be altered following 3 weeks of incubation with 25μM H_2_O_2_ (Figure 4B). The oxidative stress level was reflected in a reduced GSH/GSSG ratio rather than intracellular hydrogen peroxide level (Figure 3C–D). The protein carbonyl content was slightly increased but not significant when compared to controls (Table 1). It is likely that long term oxidative stress leads to cellular adaptation to this stressor resulting in small changes in epigenetic parameters compared to the acute situation.

### TET activity and DNA methylation under oxidative stress

The activity of TET protein, another member of the family of Fe (II) and αKG dependant dioxygenase was also compromised by oxidative stress. Initially global DNA methylation level was investigated in BEAS-2B cells that were exposed to 150μM H_2_O_2_ for 3 days that yielded a 50% cell survival rate. Global genomic DNA 5-mC and 5-hmC level was then subjected to a DNA ELISA assay to assess the relative levels of these two marks. The global 5-mC level increased from 5.5 percent (control) to 7 percent in H_2_O_2_ exposed cells (Figure 4A) and this increase was statistically significant. The cellular oxidative stress level was again measured by DCF imaging, GSH/GSSG ratio and protein carbonyl content (Figure 4B–C, Table 1). Unfortunately, 5-hmC levels were below detection limit of the DNA ELISA assay. To study the direct effect of oxidative stress on TET protein, transgenic V79 Chinese hamster lung epithelial cell line (G12) with a single copy of *Escherichia coli* guanine-hypoxanthine phosphoribosyltransferase (*gpt*) gene was utilized as a model system. Primer sets were designed to cover the entire *gpt* gene and they were named B, F, A from 5′ to 3′. Each primer set covers about a 70bp region that contains 6 to 8 CpG sites. Antibody specific to 5-hmC was used to immunoprecipitate fragmented DNA containing 5-hmC bases. Quantitative real-time PCR revealed a decrease of 5-hmC base in *gpt* DNA in H_2_O_2_ exposed cells when compared to that in control DNA (Figure 4D) which was consistent with the lowered activity of TET protein in H_2_O_2_ exposed cells when compared to that in control (Figure 4E).

## Discussion

The discovery of Fe (II) and αKG dependent dioxygenases including histone demethylases and TET proteins has provided insight into the mechanisms of how oxidative stress could transiently affect the epigenetic program in cells. Oxidative stress blocked the reduction Fe (III) back to Fe (II) causing less regeneration of active enzyme. We provided evidence to support this notion in the current study, where H_2_O_2_ impaired histone demethylase activity and excessive Fe (II) or ascorbate (Figure 2) can rescue this impairment.

Reducing agents (such as ascorbate) readily donate electrons to another substance and thereby fill up unoccupied electron levels of oxidants [24]. Iron as an electropositive elemental metal, is also a good reducing agent and thus plays an important role in enzyme mediated reactions [24]. The enzyme reaction directly leads to a transition of Fe (II) to Fe (III) that deactivates the enzyme. ROS competes with Fe (III) as additional electron acceptors leading to reduced regeneration of active enzyme [24], which is Fe (II) and αKG dependent dioxygenases.

H3K4me3 appeared to be affected most by oxidative stress in our *in vitro* tissue culture system. This may be due to the fact that this is a rather small modification present in cellular H3 (< 1%) and thus is more dynamically altered by various stressors [25–27]. The large changes in H3K4me3 have been reproducible not only in this study but also in our previous studies [28].

The current work emphasizes the importance of reduced ascorbate in attenuating transient changes in histone demethylase activity and epigenetic marks that depend on these enzymes. Most commonly employed tissue culture systems do not mimic the physiological oxygen tension or physiological ascorbate level (ambient oxygen level and minimum ascorbate supplied by serum added into culture media), rendering the dioxygenases very susceptible to oxidative stress [2]. A constant supplement of ascorbate or other ROS scavengers such as vitamin E or epigallocatechin gallate (EGCG) is not favorable because these scavengers can become pro-oxidant inducing very high levels of oxidative stress resulting in senescence or apoptosis [29]. Unfortunately, we don’t have an ideal alternative *in vitro* system available which allows ascorbate to constantly be present at physiological levels. Some of the epigenetic effects observed in tissue culture following oxidative stress might not occur *in vivo* given the protective effect of high levels of ascorbate.

Another interesting phenomenon observed in this study is the temporary decrease in global acetylation level which returned to normal levels in the case of long term oxidative stress (Figure 1 and 3). The reduction of global histone acetylation in short term oxidative stress might be due to an immediate increase of class I/II HDAC activity. Increase of class I/II HDAC activity under oxidative stress, especially HDAC3 that is responsible for global histone deacetylation [30], has been reported previously [31, 32], the mechanism remains unknown. On the other hand, other HDAC such as HDAC4 have been reported to be susceptible to oxidation and loss of their activity [33], which might contribute to a loss of total class I/II HDAC activity in the chronic exposure scenario. Class III HDAC (Sirtuin NAD+-dependent family of protein deacetylases) has been hypothesized to be up regulated under oxidative stress because NAD+ levels increase in the mitochondria under oxidative stress conditions [1], however, no direct evidence supporting this notion has been reported thus far. Only SIRT3 has been found required for the calorie restriction-mediated prevention of oxidative stress via deacetylating IDH2 [33]. The activation of SIRT3 was rather through calorie restriction but not oxidative stress, and the activation was due to increased protein level of SIRT3 rather than enzyme activities [34]. Here in this study, we found no direct evidence of increased activity of class III HDAC under oxidative stress, which is consistent with previous reports.

## Conclusion

Fe (II) and αKG dependent dioxygenases activity is reduced in the presence of oxidative stress where addition of Fe (II) or ascorbate can prevent this effect. Class I/II HDAC instead of class III HDAC activity was elevated under short-term oxidative stress, which led to a reduction of global histone acetylation.

## Figures and Tables

**Table 1:** carbonylated protein concentration in hydrogen peroxide exposed cells

H_2_O_2_ (μM)	Exposure time	carbonylated protein (nmole/mg protein) *
0		6.10±0.12
250	3 hours	9.24±0.32
150	3 days	8.32±0.65
25	3 weeks	6.40±0.51

* average ± standard deviation

## Box

- Oxidative stress impairs Fe (II) and αKG dependent dioxygenases activity
- Excessive Fe (II) or ascorbate can prevent the reduction in activity of Fe (II) and αKG dependent dioxygenases *in vitro*
- Class I/II HDAC instead of class III HDAC activity was elevated under short-term oxidative stress, which led to a reduction of global histone acetylation.
